# What do we know about how children and adolescents conceptualise violence? A systematic review and meta-synthesis of qualitative studies from sub-Saharan Africa

**DOI:** 10.1371/journal.pone.0304240

**Published:** 2024-07-05

**Authors:** Ellen Turner, Susan A. Kelly, Emily Eldred, Katrina Bouzanis, Anne Gatuguta, Manuela Balliet, Shelley Lees, Karen Devries

**Affiliations:** 1 Faculty of Public Health and Policy, London School of Hygiene and Tropical Medicine, London, United Kingdom; 2 National Institute of Teaching, Silverstone, United Kingdom; 3 Mwanza Intervention Trials Unit, National Institute of Medical Research, Mwanza, Tanzania; 4 Faculty of Epidemiology and Population Health, London School of Hygiene and Tropical Medicine, London, United Kingdom; 5 International Federation on Ageing, Toronto, Canada; 6 Global Health Office, McMaster University, Hamilton, Canada; 7 Department of Global Health and Infection, Brighton and Sussex Medical School, University of Sussex, Brighton, United Kingdom; National Institute of Public Health: Instituto Nacional de Salud Publica, MEXICO

## Abstract

**Background:**

Half of the world’s children experience violence every year, but the meaning of violence is not universally agreed. We may therefore risk failing to measure, and address, the acts that matter most to children and adolescents. In this paper, we describe and synthesise evidence on how children and adolescents in sub-Saharan Africa conceptualise different behavioural acts which are deemed violence in childhood under WHO and UN CRC definitions.

**Methods and findings:**

We conducted a systematic review of qualitative studies. We searched PsychINFO, CINAHL, Embase, Global Health, Medline and ERIC for all publications released prior to March 2023. 30 papers met inclusion criteria. We synthesised primary data from children and adolescents and drew upon theoretical and contextual interpretations of authors of included studies. Only 12 of more than 45 sub-Saharan African countries were represented with relevant research. Of the 30 included papers, 25 came from three countries: South Africa, Uganda and Ghana. Only 10 of 30 papers reported data from young children (pre-adolescence), and 18 of 30 papers primarily focused on sexual violence. 14 studies used child friendly and/or participatory methods. From this limited evidence, we identified six overarching themes in how children and adolescents conceptualised their experiences of acts internationally recognised as violence: 1) adults abusing or neglecting responsibility; 2) sexual violence from peers, family and community members; 3) violence in established intimate relationships; 4) emotional violence surrounding sex from peers and community members; 5) fighting and beating between peers; 6) street and community dangers. No studies meeting our inclusion criteria specifically examined children or adolescents’ conceptualisations of homophobic or transphobic violence; violence against children with disabilities; boys’ experiences of sexual violence from male perpetrators; trafficking, modern slavery or conflict; child labour; or female genital mutilation. We found that three dimensions were important in how children and adolescents constructed conceptualisations of violence: their age, relationship to the perpetrator, and the physical location of acts they had experienced. These dimensions were interrelated and gendered.

**Conclusion:**

The current limited evidence base suggests children and adolescents’ conceptualisations of violence overlapped with, but were also distinct from, the WHO and UNCRC definitions of violence. Currently international survey tools focus on measuring types and frequencies of particular acts and neglect to focus on children’s understandings of those acts. Relationship to perpetrator, age of child, physical location are all important in how children conceptualise their experiences of acts internationally recognised as violence, and therefore might be important for their health and social outcomes. Those developing measures should account for these dimensions when developing items for testing.

## 1. Introduction

Physical, sexual or emotional violence is experienced by more than 1 billion, or 50%, of the world’s children every year [1]. Violence during childhood has a negative impact on brain development [2], and well-documented adverse health and social consequences, including increased risk of later mental health disorders [3], substance use [3], obesity [4], poor academic outcomes [5] and suicidal behaviour [6]. Since the landmark World Report on Violence against Children [7], this issue has increasingly become the focus of global aid and policy agendas. Commitments to reduce violence in childhood now feature in Sustainable Development Goals (SDGs) 4 (Quality Education), 5 (Gender Equality) and 16 (Peace, Justice and Strong Institutions).

Sub-Saharan Africa (SSA) has the youngest population in the world [8]. Estimated prevalence of past-year violence against children aged 2–14 years in the region exceeds 80% [1], and SSA regions have high lifetime prevalence of intimate partner violence against women and girls globally [9,10], although evidence is incomplete [11,12]. Several SSA countries are also pioneering new understanding of the epidemiology of violence and how to implement effective violence prevention. Fifteen national governments have conducted Violence Against Children Surveys (VACS) to date (out of 23 countries globally) [13]. One third of the 38 ‘Pathfinder’ countries, which have made public commitments to national violence reduction action plans, are in SSA [14]. Recent systematic reviews highlight the breadth of evidence of the efficacy of interventions to prevent violence against children, and violence against women, in SSA, which outpaces other global regions in some areas. This includes, for example, adolescent intimate partner violence prevention, for which there is a greater breadth of evidence than in other low- and middle-income country (LMIC) settings globally [15,16]. For interventions to prevent adolescent IPV, more than half of the evidence from trials comes from SSA [17].

However, despite this significant interest in research, it is unclear whether the tools currently being used to measure prevalence of children’s experiences of violence in SSA resonate with how children themselves conceptualise their experiences. Much of the international epidemiological research on violence, to establish prevalence and the effectiveness of various prevention interventions [18,19], measures experience of violent acts aligning with international definitions [20–23]. The landmark World Report on Violence and Health describes the two commonly cited definitions [7]: One from the WHO describes violence against a child as, ‘the intentional use of physical force or power, threatened or actual, against a child, by an individual or group, that either results in or has a high likelihood of resulting in actual or potential harm to the child’s health, survival, development or dignity’ [24]. The second is from the UN Convention on Rights of the Child and focuses more on categorisations of violence, including: ‘all forms of physical or mental violence, injury and abuse, neglect or negligent treatment, maltreatment or exploitation, including sexual abuse’ [25]. Tools informed by these definitions follow best practice in epidemiological research by asking about specific acts (for example, ‘have you ever been hit’) to ensure comparability and avoid subjective interpretations of what constitutes violence.

Yet across diverse settings, respondents may not consider some of these behavioural acts to be ‘violence’, and, further, important experiences that are considered ‘violence’ in that setting, may be missed [26–28]. For instance, a study reviewing the content validity of a widely used tool, ICAST-C, in three different settings found that some violence questions were considered to be less relevant or had different interpretations across the contexts [29]. A recent systematic review examining children’s self-reported measures of violence, found that the majority of the commonly used measures have sufficient psychometric properties, but subsequent pilot testing in different settings is needed for content validity, and often results are not published [30].

Although studies have begun to examine child-centred tools [31], we are not aware of any widely used survey measures for adolescents that have systematically included adolescents’ own conceptualisations of violence in the instrument development process [30], or that have included the perspectives of local populations of any age group, in sub-Saharan Africa. Similarly, we are not aware of any widely used survey instruments that have been designed to measure the experiences of children below adolescent age. This is especially important considering that the epidemiology of violence against children varies markedly by age [32]; the experiences of adolescents are unlikely to be the same as younger children.

Lack of incorporation of children and adolescents’ views on the violence perpetrated against them in international public health measurements of violence risks misinterpretation or omission of important aspects relevant to determining both the prevalence of violence, and our understanding of how experiences of violence relate to poor health and social outcomes. Lack of local validity may also negatively impact uptake of study results in different contexts, particularly where concepts are considered to reflect imposed cultural values. Some emerging scholarship from sub-Saharan Africa highlights how some widely referenced ideas of ‘violence’ and children’s right to be free of violence may be perceived as foreign and detrimental to positive child development [33–35].

In this paper, we aim to understand what existing evidence tells us about how children and adolescents in SSA settings conceptualise different behavioural acts deemed to be violence under international definitions of violence in childhood. There is a certain circularity in examining what we know about children and adolescents’ conceptualisations of violence when they themselves might not use this term [36]. We address this by employing three sub-questions. First, we summarise the geographical coverage of studies, the ages of children and adolescents included, and the forms and/or acts identified as violence per international definitions and by study authors. Second, we synthesise data from included studies to explore how children and adolescents conceptualise their experiences of acts internationally defined as violence. Third, we use data from included studies to suggest a model for how children and adolescents construct conceptualisations of violence.

We draw on work conducted across a range of disciplines, such as anthropology, sociology, education, childhood studies, and social epidemiology, which use theoretical frameworks for understanding how violence is conceptualised [37–41], and summarise our findings and their relevance for international public health audiences.

## 2. Methods

We conducted a systematic review of qualitative studies and synthesised data using a combination of meta-ethnographic [42,43] and grounded theory [44] approaches. The protocol for the review is registered on PROSPERO: CRD42018103138. One significant amendment included narrowing the focus from including both community member adults and children to focusing on children and adolescents only, due to the large size of the review. The search was first conducted in 2020 and updated in 2023, using the same team and steps. Reporting of the review follows PRISMA guidance (see S1 Appendix).

### Inclusion criteria

We included studies according to the following criteria: 1) primary qualitative data, which could include qualitative or mixed-methods studies; 2) studies conducted in sub-Saharan Africa, or where data from sub-Saharan African countries was reported separately; 3) child or adolescent participants, where = >50% of the participants were under 18 years, or where findings were clearly reported separately for child or adolescent participants; 4) studies that included children and/or adolescents’ conceptualisations of violence or of acts that fall under the WHO and UNCRC definitions of violence [24]; 5) peer-reviewed studies. For criteria 4, we defined this as: a) papers with an explicit focus on how children and/or adolescents conceptualise violence, or acts covered by international definitions of violence, searching for language such as definitions, perceptions, conceptualisations, understandings, and interpretations, and/or; b) papers which included in the abstract an example of children or adolescents’ conceptualisations of acts covered by WHO and UNCRC definitions of violence, even if the paper did not explicitly focus on this (for example, ‘boys felt […] this, in their minds, did not constitute rape’ [45]). Studies that did not meet these criteria were excluded. We did not restrict by date, and all publications released prior to the dates of screening were eligible for inclusion.

### Search strategy and screening

**Fig 1:**
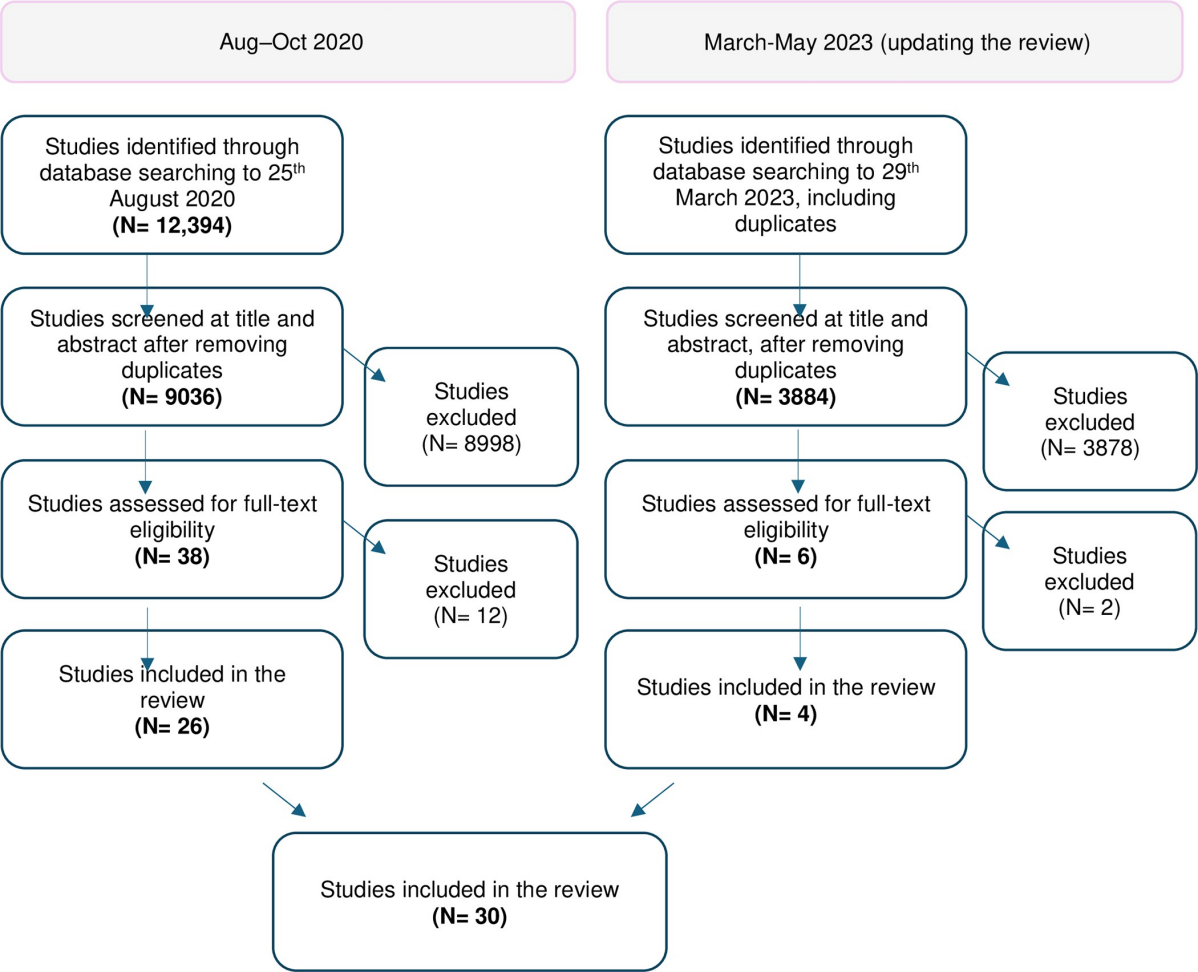
Study selection process.

We searched PsychINFO, CINAHL, Embase, Global Health, Medline and ERIC for all publications released prior to 25^th^ August 2020, and repeated the searches on 29^th^ March 2023. We used database specific terms related to: violence and abuse, children, childhood and adolescence, sub-Saharan Africa, and qualitative methods (see S2 Appendix). We did not restrict by language or date of publication. Studies were organised using Covidence software [46]. We completed the process of study selection once in 2020, and repeated it in full for the new searches conducted in 2023. Study selection is mapped in Fig 1.

The process of title and abstract screening involved multiple stages, due to the large volume of abstracts generated in our search. First (1), six reviewers [KB, MB, EE, AG, SK, ET] independently applied inclusion criteria to titles and abstracts. This process was supported by regular team meetings for comparison and standardisation, where common dilemmas were discussed and resolved, supported by example papers. Any papers that were not deemed clear in this process were tagged by these reviewers with ‘awaiting classification’ in the Covidence platform for a second round of consideration. This yielded 459 abstracts (and an additional 52 in 2023) for further consideration. Next (2), these 459 (in 2020) and 52 (in 2023) underwent a second round of screening, which included a team of reviewers [KB, ET in 2020; ET, SK, EE in 2023] applying the more detailed inclusion criteria described above for criterion 4 (4a and 4b). While dual coding was not applied at this stage, this process was supported by regular standardisation meetings. Any papers that continued to be deemed unclear were left flagged for collaborative resolution. Finally (3), these papers for collaborative resolution (49 in 2020; 12 in 2023) were considered on an individual basis, wherein two or three reviewers (KB and ET in 2020; ET, SK and EE in 2023) discussed each abstract to reach an agreement. This process was supported by dilemmas and resolutions being shared with KD and SL for input, supported by examples. Full texts were reviewed independently by KB, MB, EE, AG, SK, ET, and all full texts were reviewed a second time by ET.

### Data extraction and quality assessment

Six reviewers extracted information on: study authors’ affiliations, participants, methods, types of violence the paper focussed on, and context of the study using a standardised proforma, checked by EE and ET. We separately extracted primary qualitative data and authors’ interpretations and conclusions using NVivo software [47] for synthesis. For included studies where some participants were over 18 years, we only coded primary qualitative data for participants 18 years or under, therefore including only one additional year of adolescence beyond the WHO definition of a child (under 18 years). This was due to the review aiming to capture the views of children and adolescents, but not those over the international age of majority.

Quality was assessed with an adapted version of the Critical Appraisal Skills Programme (CASP) checklist [48], also used in other meta-ethnographic studies [49–51] (see S3 Appendix; adaptations outlined in Box 1). Quality appraisal was conducted independently by the six reviewers and involved regular discussions. We did not exclude any studies for quality, due to lack of consensus around doing so [51–54]. However, we weighted our synthesis for quality, adapting approaches used elsewhere [55], and also found that lower quality studies contributed less to the synthesis [56–58].

**Box 1. Quality assessment tool: Amendments made to the CASP quality checklist for qualitative research**

We amended the CASP quality checklist for areas of focus relevant for understanding children and adolescents’ conceptualisations of violence. These included: 1) use of social theory; 2) attention to study context; 3) use of child-friendly and participatory approaches in data collection and/or interpretation; 4) author reflexivity about their positioning and how findings were interpreted; 5) backing up findings with raw data. Finally, (6), due to the ethically challenging nature of violence research with children and adolescents, we also added additional focus on ethical considerations and child protection referral processes. For (6), to account for differences in disciplinary approaches to violence research, we extracted and critically appraised data on a) child protection referral mechanisms, and/or b) author reflexivity about handling ethically challenging situations.

### Descriptive analysis

For our first question, we describe the geographical coverage of studies; children and adolescents’ age and gender; type of violence predominantly examined, as described by the authors and international definitions; and quality descriptors. We summarise the number of studies in each category and use this to highlight gaps in the current knowledge base.

### Synthesis

We draw on meta-ethnographic approaches [43,49,58] employing Schutz’s [59] notion of first and second order constructs: First order constructs relating to the primary data from research participants, and second order constructs referring to authors’ interpretations and conclusions. SSA covers a vast and diverse area, and our sample does not represent all children and adolescents’ views across the region. We therefore did not seek consistency in conceptualisations, and instead identify trends and highlight where there is particular heterogeneity in the data. To understand how children and adolescents conceptualise acts internationally defined as violence (Question 2), we first conducted a secondary analysis and synthesis of the first order constructs reported across the 30 studies. So doing, we sought to prioritise children and adolescents’ own language and interpretations, and to retain the complexity of the topic by examining distinctions between children and adolescents’ data and others’ interpretations [60]. We then used second order constructs to check for alignment with identified themes. To explore how children and adolescents construct conceptualisations of violence (Question 3) we reversed this order, and first synthesised second order constructs pertaining to authors’ contextualised theorisations of how children and adolescents construct conceptualisations of violence, then examined alignment with first order constructs. We generated third order constructs, which are the interpretations and conclusions of the synthesis of included studies [49] (and the finding of this review), through these two layers of analysis.

To develop themes, we drew on the method outlined by Eaves [44], synthesising various grounded theory approaches [61–63]. This involved seven steps: first line-by-line *in vivo* coding, wherein the participants’ or authors’ own words were used to code sections of data; second, grouping together similarly coded sections; third, using the constant comparison method to identify relationships between codes, thus identifying categories; fourth, examining relationships between these categories to identify core or overarching categories, and sub-categories; fifth, we developed ‘mini-theories’ that through the iterative process of analysis, memo-writing, and discussion, we (sixth) developed into an explanatory theoretical framework. Our seventh step was to highlight and interrogate contradictions [49]. These steps were not linear but iterative and cyclical. Four authors (KB, EE, SK, ET) conducted the coding with regular discussion and led by the lead author (ET), and the evolving framework of categories and theory was routinely discussed with two further authors (KD and SL). Due to the heterogeneity of children and adolescents’ views, themes were topical, around which children and adolescents could hold different views (for example, ‘adults abusing or neglecting responsibility’). Inclusion of data under a theme therefore does not suggest a straightforward relationship with the theme, but shows that the data considered this topic.

For each theme, we have delineated between *strong* and *supporting* evidence [55]. Evidence was strong if: the theme was discussed in detail in a paper (versus emerging secondarily or partially); and if the study had a medium- or high-quality ranking, versus a lower ranking. Drawing on meta-ethnographic approaches used elsewhere [49], we also examined contradictions in the data, and incorporated these into the generation of third-order constructs.

## 3. Results

### Description of studies

Thirty papers were included in our synthesis (see Table 1). Participants’ ages ranged from 6 to 22 years, with the majority of studies (N = 20) conducted with adolescents aged 13 years and older. Five studies were conducted with children ranging in ages between 7 and 13 exclusively, and five studies included a mix of children, adolescents and youth ranging in ages between 6 and 22 years. The studies drew on data from 12 countries: South Africa (N = 14), Uganda (N = 7), Ghana (N = 5), Burkina Faso (N = 1), DRC (N = 1), Ethiopia (N = 1), Kenya (N = 1), Lesotho (N = 1), Malawi (N = 1), Mozambique (N = 1), Nigeria (N = 1), and Tanzania (N = 1), with at least 38 countries across SSA not represented in the data sample. Studies were conducted in a range of settings, however with more studies including evidence from urban or peri-urban (N = 25) than rural areas (N = 12), or refugee settlements (N = 1).

**Table 1:** Summary of included papers.

Paper ID (Author, Year)	Country of study	Age (years) Number	Sex	Population	Description of context (area)	Qual data collection methods (1)	Forms of violence (authors’ focus)
Addae et al., 2021 [28]	Ghana	13–18 (N = 56)	Mixed	Junior and senior high-school	(Upper West region of Ghana)	FGDs	Family violence
Ajuwon et al., 2001 [10]	Nigeria	14–21 (N = 77)	Mixed	Secondary school; shop apprentices	Urban (Ibadan)	Participatory (narrative workshops, free-listing, role-plays)	Sexual coercion
Bhana, 2008 [16]	South Africa	7–9 (N = 1*)	Female	Primary school	Urban township (KwaZulu-Natal)	Ethnography; individual interviews; group interviews; observations	Girl-on-girl violence (physical)
Birungi et al., 2011 [11]	Uganda	13–18 (N = 20)	Mixed	Secondary school	Urban (Mbarara)	FGDs	Sexual coercion
Breen et al., 2015 [15]	South Africa	8–12 (N = 24)	Mixed	Primary school	Peri-urban (nr. Cape Town)	Individual interviews	Corporal punishment
De Lange and Geldenhuys., 2012 [12]	South Africa	13–16 (N = 30)	Mixed	Secondary school	Rural (KwaZulu-Natal)	Participatory (video)	Gender-based violence in schools
De Wet, 2007 [17]	Lesotho	*Grades* 8–12 (av age 17) (N = 280)	Mixed	Secondary school	Urban, other	Mixed-methods questionnaire	School violence (verbal and physical)
Hayer, 2010 [20]	Uganda	15–18 (N = 7)	Female	Young adults	Urban, rural	Participatory (writing, drama, walks, photo); Individual interviews; FGDs	Sexual coercion
Hodes and Gittings, 2019 [9]	South Africa	14–22 (N = 66)	Male	Young adults	Urban township, rural, urban (Eastern Cape)	FGDs; Individual interviews; Participatory (drawing, life history narratives)	Sex education (including sexual coercion)
Hoss and Blokland, 2018 [8]	South Africa	13–16 (N = 32)	Female	Secondary school	Township (nr. Johannesburg)	Mixed-methods; Individual interviews; Group interviews; Questionnaire	Transactional sexual interactions
Lankster et al., 2019 [22]	South Africa	14–18 (N = 260)	Male	Secondary school	Urban (Mamelodi)	Mixed- methods; Group interviews; Surveys	Rape
Le Mat, 2016 [13]	Ethiopia	14–18 (N = 25)	Mixed	Secondary school	Urban (Addis Ababa)	Individual interviews, FGDs	Sexual violence in school
Mashia et al., 2019 [23]	South Africa	14–19 (N = 10)	Mixed	Young adults attending healthcare clinics	Urban, rural, semi-urban (One district)	Individual interviews	Peer pressure and sexual coercion
Makongoza and Nduna, 2021 [7]	South Africa	15–20 (N = 7)	Female	Young women	Urban (Soweto)	FGDs	Intimate partner violence
Mayeza and Bhana, 2020 [1]	South Africa	10–14 (N = 35)	Male	Primary school	Urban township (KwaZulu-Natal)	FGDs	Boys’ violence in school
Mayeza and Bhana, 2020 [3]	South Africa	10–13 (N = 27)	Male	Primary school	Urban	FGDs	Bullying in school
Moore et al., 2012 [21]	Burkina Faso, Ghana; Malawi, Uganda	12–19 (N = 195)	Male	Young adults (in-school and out-of-school)	Urban, rural	Mixed-methods; Surveys; Individual interviews	Unwanted sexual experiences
Muhanguzi, 2011 [14]	Uganda	14–19 (N = 368)	Mixed	Secondary school	(Central and Western Uganda)	Observations; Individual interviews; FGDs	Gendered sexual vulnerability
Mulumeoderhwa and Harris, 2015 [4]	Democratic Republic of Congo	16–20 (N = 56)	Mixed	Secondary school	Urban, rural (South Kivu)	FGD; Individual interviews	Forced sex, rape and sexual exploitation
Nyangoma et al., 2019 [25]	Uganda	6–17 (N = 43)	Mixed	Sexual abuse cases reported to police	Three post-conflict districts (Northern Uganda)	Individual interviews	Child sexual abuse
Parkes, 2007 [19]	South Africa	8–13 (N = 36)	Mixed	Primary school	Urban township (Cape Town)	Ethnography; Participatory (drama, music, art); Individual interviews; FGDs	Neighbourhood violence
Parkes, 2007 [2]	South Africa	8–13 (N = 36)	Mixed	Primary school	Urban township (Cape Town)	Ethnography; Participatory (drama, games, art); Individual interviews; FGDs	Neighbourhood violence
Parkes et al., 2016 [29]	Kenya, Ghana, Mozambique	8–17 (N = 216)	Female	In school	Rural, semi-urban (Coast Province, Kenya; Northern Ghana; Southern Mozambique)	Mixed-methods; Survey; FGDs	Sexual relationships and violence
Quarshie et al., 2020 [18]	Ghana	15–20 (N = 36)	Mixed	In-school; street-connected	(Greater Accra region)	Individual interviews	Self-harm
Ruzibiza et al., 2021 [27]	Uganda	13–19 (N = 60)	Mixed	Youth centres, schools, streets and trading centres	Refugee settlement (Southern Uganda)	Ethnography; IDIs; FGDs; participant observation	Transactional sex ; parental neglect
Sommer et al., 2013 [26]	Tanzania	16–19 (N = 160)	Male	Young adults (in-school and out-of-school)	Rural, urban (Kilimanjaro)	Observation; Participatory (stories, discussions); Individual interviews	Gender-based violence, family and peer violence
Strebel et al., 2013 [24]	South Africa	15–18 (N = n/a)	Mixed	Young adults in disadvantaged communities	Unspecified (Cape Town and Southern Cape)	FGDs	Transactional and intergenerational sexual relationships
Twum-Danso, 2013 [30]	Ghana	10–16 (N = 23)	Mixed	School children in public and private schools	Rural, urban (Greater Accra and Eastern regions)	Mixed-methods; Individual interviews; Diaries; Questionnaire	Physical punishment from caregivers
Wagman et al., 2009 [6]	Uganda	15–17 (N = 52)	Female	Young sexually active adults (pregnant and never pregnant)	Rural (Rakai District)	Individual interviews; FGDs	Sexual coercion
Wood et al., 1998 [5]	South Africa	14–18 (N = 24)	Female	Young adults (pregnant, attending obstetric unit)	Township, peri-urban (nr Cape Town)	Individual interviews	Violence and coercion in sexual relationships

(1) FGD—focus group discussions; Interviews–in-depth interviews, semi-structured interviews.

The majority of studies focussed primarily on sexual violence (N = 16), with the remaining papers focusing primarily on violence in intimate relationships (N = 2), emotional or physical violence between peers (N = 4), gender-based violence (N = 2), physical punishment (N = 2), neighbourhood violence (N = 2), family violence (N = 1), and self-harm (N = 1).

Studies varied in how they asked children and adolescents about violence, although specific questions were most often not reported. In most studies, researchers introduced a concept (such as ‘punishment’, ‘sexual coercion’) and asked children and/or adolescents to describe what acts or experiences they associated with the concept (N = 15); roughly a quarter of studies involved researchers asking about specific acts, covered by international definitions of violence, and asked them to describe their experiences (such as ‘having sex that was unwanted’ or ‘relationships with taxi drivers’) (N = 8); and in roughly a quarter researchers asked about other topics (e.g. sex, family relationships, feelings of safety) and conceptualisations of violence emerged during discussions (N = 8).

#### Limitations in study coverage

At least 37 countries across sub-Sahara Africa were not represented in the studies, with particular gaps in Central Africa. South Africa, Ghana and Uganda, which have English as the predominant language of government, are Commonwealth countries with more than 25 years of independence from colonial systems and are among the top 15 African countries with the highest GDPs, accounted for 26 of the 30 included studies. Such characteristics may have led to the increased number of studies in these SSA nations to the exclusion of other SSA countries. There was more evidence from urban and peri-urban settings (N = 25), with fewer studies including evidence from rural areas (N = 12), and only one from a refugee settlement. Most studies focused on the views of adolescents (N = 25), with some studies including the views of younger children (N = 10), and none that we ranked as medium or high quality focused on the views of young children (of pre-adolescent age) specifically. Therefore, we currently know very little about how young children conceptualise violence.

No studies in our sample explicitly aimed to understand how children and adolescents perceived violence as a concept. No studies specifically focused on neglect or emotional violence, and few studies focused on physical violence (although data emerged from various papers about these forms of violence, as highlighted in our thematic summary). Themes for non-sexual forms of violence did not always reach thematic saturation due to limited sample size. Self-directed violence was the focus of one paper, but we did not list this is as a separate theme due to small sample size. We did not find any studies looking at violence specifically linked to stigma, such as homophobic violence and violence against children and adolescents with disabilities; boys’ experiences of sexual violence from male perpetrators; experiences of children and adolescents affected by trafficking, modern slavery, or conflict; child labour; and female genital mutilation.

#### Study quality

Quality varied across our 30 included studies, shown in Table 2. Most studies were ranked as partially- or well-contextualised (N = 26) or were rooted in theoretical frameworks (N = 18). Most studies included substantial raw data to back up findings and details on how data were analysed, and most included authors affiliated with organisations located inside the study country. However, there were limitations to study quality. Only fourteen papers described using child friendly (N = 7) or participatory (N = 7) approaches to data collection. Sixteen papers did not explicitly mention child-friendly, participatory or sensitive approaches as an aspect of study design. Only six studies describe having either a referral protocol in place or a link to follow-up services, and one study pertaining to two papers mentions being linked to a local violence response organisation. Twenty-two studies did not mention offering follow-up services for participants; however, 10 were linked to a larger study and may have included these details elsewhere. No study described how these protocols worked in practice or included authors’ reflexive accounts of handling disclosures of violence.

**Table 2:** Quality of included studies.

Selected quality markers	Partially or substantially included in paper (Total N = 30)
**Use of social theory**	N = 18
**Attention to study context**	N = 26
**Authors from study country**	N = 22
**Child-friendly and/or participatory approaches**	N = 14 *Child-friendly N = 7* *Participatory N = 7*
**Child protection referral mechanisms in place**	N = 6 *(However an additional two were linked to violence response organisation N = 2)*
**Author reflexivity about handling challenging cases of violence disclosure**	N = 0
**Descriptions of how child protection referral mechanisms functioned in practice**	N = 0
Overall medium or high ranking (included as strong or supporting evidence)	N = 25
Overall low ranking (included as supporting evidence only)	N = 5

### Synthesis of included studies

*How do children and adolescents conceptualise their experiences of acts internationally defined as violence*?. Overall children and adolescents rarely used words such as violence, abuse or maltreatment when describing acts internationally defined as violence. Children and adolescents’ views were highly heterogenous, both within and across studies, however we identified six overarching themes in how they conceptualised experiences of acts internationally recognised as violence. We highlight within each section where there was most concordance, or heterogeneity, within the data. The six themes included: 1) adults abusing or neglecting responsibility; 2) sexual violence from peers, family and community members; 3) violence in established intimate relationships; 4) emotional violence surrounding sex from peers and community members; 5) fighting and beating between peers; 6) street and community dangers. Evidence was strong overall for Themes 1, 2, 4; strength of evidence was mixed for Theme 3, according to sub-theme; and evidence was weak overall for Themes 5 and 6 (shown in Boxes 2–7).

**Box 2. Theme 1 summary evidence**

**Table:** 

Sub-theme	Strength of evidence		Illustrative examples (first order constructs)
	***First order constructs***	***Second order constructs***	
Fairness and unfairness of physical and emotional punishment	**Strong**: [3, 15, 18, 28, 30] **Supporting:** [1, 2, 7, 17, 26]	**Strong:** [2, 15, 18, 26, 28, 30] **Supporting:** [[1, 3, 17, 19]	She [my mother] beats me and I cry and I become sad (9-year-old girl, South Africa) [15] Mom will hit me when I play outside […] Because someone can catch me… and rape me also (8-year-old-girl, South Africa) [2] Physical punishment is not bad if the child is wrong but should not be done excessively. Beating a child all over the body mercilessly is not good (16-year-old girl, Ghana) [30] My mum is a very caring, strict woman who loves me very much. She cares for me a lot to the extent that she puts me in my place whenever I misbehave either by throwing insults at me or whipping me, but she has not whipped me since I turned 14 because I am now a young man (14-year-old boy, Ghana) [30] You know what happens when girls report us to teachers? The teachers punish us! The punishment is very harsh. They will shout at you and they will give you some hiding too! They hit us with a stick! But, girls are not punished for all the violence that they do to us in the yard during break (Primary school boy, South Africa) [3]
Neglect and relinquishment of caregiver responsibility	**Strong:** [18, 25, 27, 28] **Supporting:** [4, 6, 7, 11, 15, 20, 24, 26, 29, 30]	**Strong:** [18, 25, 27, 28] **Supporting:** [4, 11, 15, 20, 24, 26, 29, 30]	We are always left alone to play with other children of the neighbours and this made this man called X to defile me after he had learned that both my parents are never at home during the day. Our parents are always too busy with their work (6-year-old girl, Uganda) [25] Some of the fathers are irresponsible or don’t work – they leave home with no money for food (16-year-old boy, Tanzania) [26] I’m only a small boy, why should I be struggling as if I am a father? ([age not given] boy, Ghana) My grandmother told me she is very old and does not have money so we (orphans) should cater for ourselves... No, I didn’t want the first sex – I wanted money to buy books. If I had books, I would not have had sex (16-year-old girl, Uganda) [6]
Witnessing violent behaviour by or between caregivers	**Strong:** [26] **Supporting:** [2, 7, 15, 18, 27, 28]	**Strong:** [26] **Supporting:** [7, 19, 28]	Sometimes you will find a father…when he comes home drunk, he tries to beat the mother. And when you see that, you feel bad. So you can even stop studying and start to cry (18-year-old boy, Tanzania) [26] Occasionally, he [my father] drags us [the children] into his fight and ‘charade’ with my mother in the house. He hits and slaps me because I question him […] (16-year-old girl, Ghana) [18]
Sexual harassment by institutionally responsible adults	**Strong:** [4, 13] **Supporting:** [12, 14]	**Strong:** [13, 14] **Supporting:** [4, 6, 12, 17, 20]	Our way of wearing can induce rape. Most of our teachers are young men and newly married men […] I do not know a teacher who can resist looking at that (18-year-old girl, DRC) [4] Sometimes when students get a mark, low mark, mark decrease. Then, the teachers speak about sexual intercourse […] The students are very afraid (Adolescent girl, Ethiopia) [13] We (girls) will get difﬁculties from students, from boys. Or from male teachers. ( … ) But, if we think that, we are learning for knowledge, we have to accept whatever it is that happens to us (Adolescent girl, Ethiopia) [13]
Early marriage	**Strong:** **Supporting:** [28, 29]	**Strong:** **Supporting:** [28, 29]	Sabina: Yes we go to Action Aid and now the parents are very afraid of Action Aid. They report to police and then you are put in prison. Fazul: Now we are not seeing cases of early marriage a lot as in the past. Because we can report and then the parents are put in prison (Adolescent girls, Kenya) [29]
Non-responsive and neglectful treatment by institutionally responsible adults	**Strong:** [14] **Supporting:** [1, 2, 7, 12, 19, 23, 25]	**Strong:** [13, 14, 25] **Supporting:** [1, 2, 4, 7, 12, 19, 23]	Some teachers […] don’t see what is happening. They just stand and keep talking and laughing. They don’t watch the children being hurt… (Young adolescent boy, South Africa) [1] Such things sometimes you don’t tell the teachers... Even if you report, they do nothing, they say you are just faking it or creating stories... I have not seen any one being punished for that (Adolescent girls FGD, Uganda) [14] [The police] must be active there in the coloured areas also ... because they’re very quick in the white areas but they’re not so quick here (13-year-old boy, South Africa) [2]

**Box 3. Theme 2 summary evidence**

**Table:** 

**Theme 2. *Forcing a girl to sleep with her is rape*: Sexual violence from peers, family and community members**			
**Sub-theme**	**Strength of evidence**		**Illustrative examples (first order constructs)**
	***First order constructs***	***Second order constructs***	
**Sexual harassment, coercion and forced sex by peers**	**Strong:** [4, 6, 10–14]	**Strong:** [4–6, 9–14]	If one boy loves any girl, he pleases them [referring to insisting on having sex]. He really really pleases them. So she can’t stop him (Adolescent girl, Ethiopia) [13]
		
	**Supporting:**	**Supporting:** [8, 17, 20–222]	I was in class copying notes and a boy came up, like they always do, and touched me on my breasts— something which did not make me feel good (17-year-old girl, Uganda) [6]
	[5, 9, 17, 18, 20–22, 25]		
			Together they determine a plan to ‘teach that arrogant girl a lesson’ […] Bayo and his friends ambush and overpower her […] Bayo informs her that he would not have raped her if she had complied with his request for sex (Female adolescents, Nigeria) [10]
		
			Boy 1: If the girl doesn’t want to, I would watch porn with her. I can touch her all over her body
			Boy 2: I would make her drink ‘silver bullet’ [a branded, locally-available sexual performance aid]
			Boy 3: I can drug her, because I don’t have any other way
			Boy 4: That’s rape dude
			(Male adolescents, South Africa) [9]
		
			‘[Girls] see women on TV with short skirts and so in Tanzania the girls try to copy, so they are raped’ (Adolescent boy, Tanzania) [26]
		
**Forced sex and harassment by (older) males and community members**	**Strong:** [6, 25]	**Strong:** [6, 25]	I was sent to the market […] and on my way back, I met two boys [they] carried me by force to the bush and started raping me. When I heard people passing, I shouted loudly at once. They came and rescued me. I reported to my father […] These boys were arrested very early in the morning of the following day. They are now in jail (15-year-old girl, Uganda) [25]
	
	p>**Supporting:** [2, 4, 10, 11, 26, 29]	**Supporting:** [2, 4, 5, 11, 14, 19, 20, 29]	
	
**Sexual violence within families**	**Strong:** [25]	**Strong:** [10, 25]	I didn’t want [my stepfather] to sleep with me again, so I drank a [small] bottle of ‘parazone’ [bleach]. I wanted him to see that I hated what he was doing to me […] (18-year-old girl, Ghana) [18]
		
	**Supporting:** [5, 6, 10, 11, 18, 28]	**Supporting:** [5, 6, 288]	I share a house with a boy (12 years) who is related to my stepfather […] he used force and had sexual intercourse with me. I reported him to my mother and there was a family gathering […] He was reported to police but the case was handled at family level since there was a need to cleanse the curse (7-year-old girl, Uganda) [25]
		
**Exchanging gifts and basic needs for sex**	**Strong:** [4, 6, 8, 11, 24, 25, 27, 29]	**Strong:** [2, 4, 6, 8, 11, 24, 25, 27, 29]	He used to tell me “I love you and I will give you money.” [I had my first sex with him] because I wanted snacks very much (17-year-old girl, Uganda) [6]
		
	**Supporting:** [2, 7, 10, 20, 22]	**Supporting:** [5, 7, 20, 21]	The girl has no right to say ‘no’ to sex in the house of the sugar daddy (Adolescent girls, South Africa) [8]
		
			We often experience rape cases here […] you ﬁnd a married man who is 40 years old perpetrates rape because he has got money. When he meets a girl in the community who is ﬂourishing [...] she is going to love him because of money (18-year-old boy, DRC) [4]
		
			Girl 1: And it’s degrading. Like for us it would be degrading, degrading, degrading
			Girl 2: It’s impossible to have a ‘normal relationship’ with taxi drivers, it’s always about sex and drugs, they only buy you a drink to overpower her to have sex with her (Adolescent girls, South Africa) [24]

**Box 4. Theme 3 summary evidence**

**Table:** 

**Theme 3. *As a woman you have no rights*: Violence in intimate relationships**			

**Sub-theme**	**Strength of evidence**		**Illustrative examples (first order constructs)**
	***First order constructs***	***Second order constructs***	
**Forced and coerced sex in peer dating relationships**	**Strong:** [4–7]	**Strong:** [4–7, 9]	When I date a girl, I have an objective to have sex with her. I provide her with money or buy her soap and body cream. Then, in this circumstance, if she refuses to have sex with me, I can break up with her and look for another girl who can give me what I am looking for (17-year-old boy, DRC) [4]
		
	**Supporting:** [9–12, 20–22]	**Supporting:** [8, 10–13, 20–22]	For adolescents, for them they just fall in love with each other, but I do not think that they are forced. The forced sex I know is between the adult and the young people. That one I know is where there is forced sex’ (Adolescent (sex not stated), Uganda) [11]
		
			[H]e told me that if you agree to having a relationship with a boy, you are committing yourself to having sex with him’ (Adolescent girl, South Africa) [5]
		
			Forcing a girl to sleep with her is rape. People must look for an agreement [to have sex]. .. . They should not live like animals. Boys and girls may have the urge to have sex, but he should not force her – that is rape (17-year-old girl, DRC) [4]
		
**Forced and coerced sex in established relationships**	**Strong:** [5, 6]	**Strong:** [5, 6]	Could you be married and refuse to do those things (have sex)? Then what are the reasons as to why you came into marriage?! (17-year-old girl, Uganda) [6]
	
	**Supporting:** [4, 7, 20]	**Supporting:**	
		[7]	
**Beating in relationships**	**Strong:** [5, 7, 26]	**Strong:** [5, 7, 26]	They don’t care, they will hit you anywhere, face and all. You would think they would at least avoid that because your parents will see the bruises and the injuries, but they don’t care (Adolescent girl [14-18 years], South Africa) [5]
		
	**Supporting:** [20]	**Supporting:**	Sometimes I let him beat me until he gives up (Adolescent girl, South Africa) [5]
		
			One day a father came from the job… After some minutes he […] started beating his wife, saying why didn’t she come and take the bag when he was arriving. So he put the family in a hard situation, and the children start having fear (16-year-old boy, Tanzania) [26]
		
**Lack of decision-making power, and choice in intimate relationships**	**Strong**: [10]	**Strong:** [5, 6]	If you are married and living in the man’s house you cannot make your own decisions; it’s the man who decides for you (17-year-old girl, Uganda) [6]
		
	**Supporting:** [5–8]	**Supporting:** [7, 8, 10, 12, 20, 26]	As a woman you have no rights. You must keep quiet and do as the man wants (Adolescent girl, South Africa) [5]
		
			[Regarding condom use, men say] ‘When I bought you a beer it didn’t come in a plastic, it comes clear’ (Adolescent girl, South Africa) [8]

**Box 5. Theme 4 summary evidence**

**Table:** 

**Theme 4: *Vulgar words*, *peer pressure and stigma*: Emotional violence surrounding sex from peers and community members**$BREAKBREAK$			
**Sub-theme**	**Strength of evidence**		**Illustrative examples (first order constructs)**
	***First order constructs***	***Second order constructs***	
**Peer pressure to have sex**	**Strong:** [5, 8, 9]	**Strong:** [5, 8, 9]	They would ask you if you have ever ‘tasted’ [penetrated] the vagina, and you would be pressured to do so, and end up having sex (Adolescent boys, South Africa) [9]
		
	**Supporting:** [6, 10, 12, 21, 23, 24]	**Supporting:** [6, 10, 12, 14, 21, 23, 24, 27]	My friends ... often tease me and say that I am not a correct guy [because I haven’t had sex] (16-year-old boy, Ghana) [21]
		
			if you want to belong to that group you end up doing it, otherwise you become isolated and nobody wants that (Adolescent girl, South Africa) [5
		
**Bullying and harassment for rejecting sex**	**Strong:** [10, 14]	**Strong:** [10, 14]	So when you refuse to love any of them they all hate you as in that they are gossiping about you. Everything they do is to embarrass you in front of your friends and even in class (Adolescent girls, Uganda) [14]
		
	**Supporting:** [5, 12]	**Supporting:** [5, 12]	Moscow and his friends decide the most appropriate time and place to ridicule her. His main aim in doing so is to ‘belittle what he could not get’. He and his friends choose a public place such as a street along a major road to commit the act. Moscow and his friends yell abuses at her, belittle her and jokingly touch her breasts and backside to embarrass her (Female students role-play, Nigeria) [10]
		
**Shame, stigma and gossip for sex, transactional sex, pregnancy and sexual abuse**	**Strong:** [5, 6, 14, 24, 25]	**Strong:** [5, 6, 14, 24, 25]	Girl 1: Hoere, hoere. [Whores]
			Girl 2: It’s like you, you’re dirty
	**Supporting:** [4, 8, 18, 22, 27, 29]	**Supporting:** [4, 8, 11, 13, 18, 29]	Girl 3: There’s a vulgar word for it, jintoe [laughter]
			Girl 4: And even parents in the community won’t want me to befriend their children because there is the perception that I will negatively influence them. That I’m wrong and naughty (Adolescent girls, South Africa) [24]
		
			It would be a big shame to be pregnant so we have not seen it in our school (12-13-year-old girl, rural Kenya) [29]
		
			Even as a boy if you tell a girl for example ‘Michella can we have a love affair and the girl just says… ’Ooh let’s go.’ Again you will start asking… [laughter] […] you are like… This girl is not serious, is easy going, may be she is sick… or wants to take my pocket money (Adolescent boys, Uganda) [14]

**Box 6. Theme 5 summary evidence**

**Table:** 

***Theme 5*: *Bigger boys… they spoil for a fight*: Fighting and beating between peers**		
**Strength of evidence**		**Illustrative examples (first order constructs)**
	***First order constructs***	***Second order constructs***	
**Physical fighting and bullying between peers**	**Strong:** [1–3]	**Strong:** [1–3]	Boy 1: I hate bullies because they take advantage of us and the other smaller boys at school
			Boy 2: I’m so scared of bigger boys… They have bigger muscles and they bully us. They forced me to give them my lunch and money, they made me starve. (Young adolescent boys, South Africa) [1]
	**Supporting:** [15–19]	**Supporting:** [16, 17, 19, 26]	** **
			I ask him, do you want to fight and if he’s willing to go argue, then I’ll take him on, ’cause I’m a person I’m scared of nobody / they look up to us probably because we’re the strongest in the class (15-year-old boy, South Africa) [19]
			** **
**Fighting and judging between girls**	**Strong:** [8, 24]	**Strong:** [8]	Pindile and her friends were talking about us and we heard them […] She is not my friend. She shouts at us and she doesn’t share lunch with us (7-9-year-old girl, South Africa) [16]
	** **	** **	
	**Supporting:** [2, 16]	**Supporting:** [2, 16, 24]	Girl 1: And then people call you names, in that community, just because you’re dating a taxi man […]
			Girl 2: Yes, you’re isolated
			(Adolescent girls, South Africa) [24]
		
**Physical punishment by peers and siblings**	**Strong:** [30]	**Strong:** [30]	If my little sister does something wrong, I usually give her a slap on the buttocks to show her that what she did was not right so that she won’t do that again next time (14-year-old girl, Ghana) [30]
	
	**Supporting:**	**Supporting:** [15]

**Box 7. Theme 6 summary evidence**

**Table:** 

**Theme 6. *Not nice to go*: Street and community dangers**			

**Strength of evidence**		**Illustrative examples (first order constructs)**
	***First order constructs***	***Second order constructs***	
**Community and gang-related violence**	**Strong:** [2, 19]	**Strong:** [2, 19]	There’s just nothing is good because on every corner there’s gangsters and we’re very scared to walk past them now and I don’t want to actually play outside… There’s nowhere a safe place to play because there’s just gangsters, gangsters – everywhere you see is gangsters’ (13-year-old girl, South Africa) [19]
			** **
	**Supporting:** [18, 26]	**Supporting:**	[People join gangs] because they want to be cool, want to be taken notice of (Adolescent boy, South Africa) [19]
		[18, 26]	
		
**Dangers for street-connected children**	**Strong:** [18]	**Strong:** [18]	[…] [T]hings were difficult, usually no food to eat, no money to buy food, no work to do, nothing. Sometimes I could be walking in the market and someone would just scream ‘juleei! [thief!]’ […] then people from nowhere would just pounce on me and beat me up, because no one knew me here, I was new’ (16-year-old boy, Accra) [18]
		
	**Supporting:**	**Supporting:**	My cousin […] told me that if I wanted to live here (in Accra) then I would have to be strong, stay out of trouble with boys I don’t know, and stick with the girls all the time and never walk alone […] That statement my cousin made is what has kept me safe to today (16-year-old girl, Accra) [18]
		
**Adult violence as protection**	**Strong:** [2, 19]	**Strong:** [2, 19]	I saw a man […] my daddy did go fetch his shotgun and so he ‘pah-pah-pah’ […] so he strike it and so throw it just to get him away (8-year-old boy, South Africa) [19]
		
	**Supporting:** [15]	**Supporting:** [26]	Boy 1: […] it’s right for the police to hit them because they were hitting the girls
			Boy 2: […] the police is also trying to protect us
			Boy 3: But sometimes the police bully the people
			(10-year-old boys, South Africa) [2]
		
			They hurt a lot of people; they kill people; the drugs that they give people kill the people. So I think it’s time that they take their lives away from them (15-year-old boy, South Africa) [19]
		
**Risks related to conflict**	**Strong:**	**Strong:**	n/a
	
	**Supporting:**	**Supporting:** [4, 25, 27, 29]	
	[27]

### Theme 1. *My caregiver does not love me or even care*: Adults abusing or neglecting responsibility

This theme was discussed by both children and adolescents with ages across studies ranging from six to 18 years. Children and adolescents often described the ways in which they were treated by adults in positions of responsibility, either caregiving or professional. They described experiences that were negative and left them feeling mistreated, either through causing physical or emotional pain, a sense of unfairness or injustice, and/or a failure of adults to live up to their expectations. These experiences could vary across different settings and for different children and adolescents, however, were connected in their narratives by the positions of authority or responsibility that adults had over them and by the expectations they had for fair and good treatment by adults.

Perceptions of neglectful or abusive treatment by adults related to *unfairness of physical and emotional punishment*, in relationship contexts where adults were not loving or supportive or when punishment was deemed unfair or excessive [64–67]. However, while some children and adolescents emphasised painful emotions [65,68], physical punishment viewed as reasonable was broadly tolerated as an everyday part of upbringing and training by many [64,65,69–71]. Children and adolescents across settings related painful or difficult experiences linked to *neglect or relinquishment of caregiver responsibility* through absence of caregiver warmth or emotional support [64,65,67,72,73] or lack of financial and resource-related support, or safety, guidance and monitoring [45,67,72–78], which at times included withholding vital care [67]. *Witnessing violent behaviour by or between caregivers*, which was a violation of children and adolescents’ expectations about responsible ways in which adults should behave, also left them feeling angry, sad, scared or disdainful [67,69,71,73,79], although was justified by some [80]. A small number of adolescents described girls being forced into *early marriage* as a form of mistreatment [67,81]. Children and adolescents also described *sexual harassment by institutionally responsible adults* [45,82–84], where children highlighted power imbalances and fear due to the perpetrators’ institutional authority. However, their views on this were highly varied, as some viewed this in terms of transactional relationships or gender norms rather than through a lens of teachers’ abuse of institutional authority [45,82]. Children and participants also described *non-responsive and neglectful treatment by institutionally responsible adults* where adults in a position of responsibility in: schools [70,82–84]; judicial systems [45,73]; nursing [85] and social work settings [73]; and police in relation to neighbourhood violence [69,71,80] had not taken adequate or satisfactory action to protect them or had treated them unfairly.

### Theme 2. *Forcing a girl to sleep with her is rape*: Sexual violence from peers, family and community members (outside of intimate partnerships)

Twenty-three papers fell within this theme with the majority of the studies including adolescents above the age of 13 years (N = 18) discussing various forms of sexual coercion and force, mostly by men/boys against girls. Age and relationship to perpetrator were key to how young people discussed sexual violence. Many boys downplayed *sexual coercion or rape between adolescent peers* and largely viewed adult males as perpetrators of sexual violence [45,82,86], while girls did not often focus on perpetrator’s age as a defining aspect of sexual violence. Adolescents described wide-ranging forms of sexual coercion, including unwanted sexual touching or comments, described more often by girls [75,82,84,87]; boys forcing a girl ‘because’ she said no [83,87–89]; harassment and humiliation for girls rejecting boys’ advances [84,87]; being drugged [86,90]; and verbal insistence [75,82]. Coercive situations were not, however, consistently defined across or within studies. Girls spoke with more consistency about acts that they felt were sexually violent [45,75,82,84], although these too were highly heterogenous. Across these studies, discussions centred around perception of male sexuality as dominant, unchangeable, or uncontrollable, while girls’ behaviours were seen as responsive to boys’ actions. A small number of boys discussed experiencing coercion from girlfriends or older women [86,91].

*Sexual assault from mostly older males in the community* was discussed less often, but was defined more clearly and consistently as rape, and was among the experiences most unequivocally viewed as violence by children and adolescents [68,71,73,79,81,88]. Survivors of sexual assault were almost always described to be girls, with a small number of boys in Uganda experiencing sexual assault or exploitation from older women in the community [73,86]. A small number of cases of *sexual violence within families* were discussed, involving unwanted touching, indecent exposure, sexual coercion or forced sex from (mostly older) male to younger female relatives [72,73,87,88], and also unequivocally viewed as violence, although more taboos were placed around this [73,87,88]. Adolescents described *exchanging sex for money and goods* at length and across many settings. This incorporated a wide range of behaviours. While girls particularly did not view this as violence, it was linked to other forms of violence: resource exchange was generally accepted within relationship norms [75,86], but could lead girls to being unable to say no [45,75,76,78,86,87]; transactional relationships with older men were heavily criticised or seen as rape by boys in DRC [45]; girls engaging in sex for money or goods were widely described to be stigmatised by peers [45,71,74,77,81,89]; and perceived to be particularly vulnerable to coercion and control from their partners [73–77,86].

### Theme 3. *As a woman you have no rights*: Violence in established intimate relationships

Studies within this theme included adolescents exclusively (N = 14) with participants ages ranging between 12 and 18 years. This theme examined adolescents’ perspectives on violence in intimate relationships, which they saw as taking place within (hetero)sexual, gendered relationship expectations. Across studies, adolescents discussed *forced and coerced sex in peer dating relationships*, generally perceiving that dating or being in a relationship with a peer involved expectations for sex, which could lead to force [45,75,76,80,87–91]. What constituted violence was not clearly defined within or across these studies, however, with both girls and boys holding differing views within and across studies. A small number of studies suggested that *forced and coerced sex in marital or cohabiting relationships* was perceived differently to dating relationships due to marital expectations, women’s lack of power and inability to say no to husbands, and fears of community perceptions [75,88]. Unmarried girls in relationships were more able to say no and negotiate sex than married girls in a study in Uganda [75]. *Beating and physical violence* within relationships was viewed by boys in Tanzania as emerging out of conflicts over gendered norms of financial provision and running a household, but were seen as inappropriate male behaviour [79]. Adolescent women in South Africa described physical beatings by their husbands in wide-ranging ways, but most often as something private and something they endured as part of a relationship [80,88]. Being in a relationship with, or accepting money from a man, was described to lead to *lack of decision-making power and choice*, particularly with regards to family planning, such as condom use or abortion, as described by mixed-gender groups of adolescents [83,87] and girls [75,77,88].

### Theme 4. *Vulgar words*, *peer pressure and stigma*: Emotional violence surrounding sex from peers and community members

Eighteen papers appeared within this theme which discussed emotional bullying and violence. The majority of the studies (N = 16) included adolescents exclusively within the age ranges of 12–18, with two papers including children and adolescents ranging in ages between 6 and 17. A common theme that emerged from children and adolescents’ narratives was the influence of peer pressure and community judgement, particularly around adolescent sexual choices and experiences. While not consistently viewed as violence, adolescents across many settings discussed painful or negative experiences of gossiping, pressure and stigma surrounding sex, and these were highly gendered. *Peer pressure to have sex* occurred often, with boys describing facing pressure from male peers to have sex or to not accept no for an answer, often linked to ideals of sexualised and dominant masculinity [87,90]. Girls also described experiencing pressure from female peers to have sex [88] or to have sex for resources [74,75,77]. Girls in a Ugandan study described how they experienced *bullying and harassment for rejecting sex* from groups of boys [84], while boys in this study simultaneously judged or harassed girls initiating or accepting sex too readily. This was also seen in a South African study [88]. Mixed-gender groups labelled boys using humiliation or force for girls who rejected them as violence or coercion [83,87]. Across varied settings, girls described facing shame, stigma, judgment, gossip, and/or social isolation from their peers for, most often, transactional sexual relationships [74,77,81], but also around girls’ sexual desires [72,84,88,89], pregnancy [81], and rape [45,75,88]. Two boys described isolation and shame for experiencing sexual exploitation by older women in Uganda [73]. Adolescents did not tend to view such shame and social stigma as violence, but it was discussed at length across these studies and in relation to painful emotions.

### Theme 5. *Bigger boys… they spoil for a fight*: Fighting and beating between peers

Twelve papers appeared within this theme with half the papers (N = 6) including the voices of children between the ages of 8 and 14 exclusively. Of the remaining six papers, five papers included adolescents between the age ranges of 13 and 18, and the sixth paper included both older children and adolescents between 10–16 years old. Some studies examined *physical fighting and bullying between peers* that occurred most often in schools [65,66,68–71,92]. This mostly involved boys and was linked to power dynamics related to age and size, with older or bigger boys seen to beat or take advantage of younger boys and have access to status and protection [69–71]. One study examined physical violence by girls in South Africa, described as being ‘learned’ from boys [92], and other young boys described girls bullying younger boys for food and for evading punishment from teachers [66]. Some girls also described *fighting and judging between girls*, with girls verbally harassing or excluding each other, as seen with younger girls [92], and among adolescent girls (closely related to Theme 4) in gossiping and judging among female peers around sex [71,74,77]. This was not widely seen as violence. In one study adolescents described using *physical punishment with peers or siblings*, mostly younger, viewed as fair punishment when in line with caregiving responsibilities [64].

### Theme 6. *Not nice to go*: Street and community dangers

Six studies elucidated children and adolescents’ perspectives on physical violence and crime occurring in their neighbourhoods. Three studies exclusively included children with ages ranging between 8 and 13, and the other three studies included adolescents ages ranging between 13 and 20. Children and adolescents’ perceptions of this violence were tied to feelings of safety and danger in their communities and mostly linked to violent community actors which they disassociated from themselves. In a South African township [69,71] children described acts of *community and gang-related violence* and crime, to which they attributed multiple meanings, viewing it both with fear, hostility, repulsion, helplessness and disempowerment, while also feeling a pull towards violence and the capital it could afford [69]. In urban Accra, Ghana [72] adolescents described *dangers due to being street-connected*, which they viewed in terms of their safety amid multifarious dangers of living on the street. In South Africa children described *justified adult violence as protection or retaliation*, by older male family members or the police, as a reaction to unjustified community violence, with a clear distinction drawn between fair and unfair violence [69,71]. Three studies examined *violence in conflict-affected settings*, such as in the aftermath of protracted and widespread conflict [45,73] or recent community conflicts [81]. Children and adolescents did not focus on the conflict but focused more on relationships and individual interpersonal violence. Burundian adolescents in a refugee setting in Uganda described a range of risks they faced in the settlement, including perceived parental neglect and sexual risks, particularly for girls, although this latter was seen to hold some positive aspects [78]. However, these adolescents focused on changes they experienced in their lives since displacement and did not discuss the conflict itself.

*What do we know about how children and adolescents construct conceptualisations of violence*?. Across the studies and themes, children and adolescents’ conceptualisations of violence were highly relational. They focused less on the acts themselves than on their emotions and overall wellbeing. We found that both children and adolescents made meaning of their experiences in relation to three main aspects: 1) relationship with perpetrator; 2) location and physical space; and 3) age. These three aspects were highly gendered in nature and were interrelated. At the heart of these conceptualisations were a range of painful emotions for children and adolescents, including anger, unfairness, sadness, pain, shame, insecurity, powerlessness, and humiliation.

#### Relationship with perpetrator

Children and adolescents’ perceptions of violence were inextricably connected to relationships within which experiences occurred, rooted in norms and expectations for relationships in particular settings. For caregiving relationships, this could be seen in the emphasis children and adolescents placed on caregivers’ failure to live up to expectations of provision and care, viewed as essential for survival and wellbeing, and core tenets of the caregiver-child relationship across many settings [45,64,67,72,73,78,79]. Children and young adolescents in one study in Ghana defined some level of physical punishment as an acceptable, even important, aspect of caregiving [64], while adolescents from across Ghana described their perception of extreme experiences of violence, such as punishment, withholding of care, and abuse of authority (‘how can I feel safe at home?’) that emerged out of caregiving norms [67]. For institutional relationships, this centred around perceptions of appropriateness of acts, or failure to act, within institutional authority [45,68–71,73,79,82,84,85]. For sexual violence, the same act was often viewed differently relating to different perpetrators [45,75,86,87], with young women in Uganda and South Africa using explicitly different terminology for forced sex from intimate partners or non-partners [75,88]. For romantic relationships, while adolescents’ relationships were negotiated in light of widespread hegemonic masculine norms and perceived dominance of male sexuality [45,79,80,86,87,89], these were also highly heterogenous. Experiences in line with WHO definitions of violence were seen by both girls and boys, within and across studies, as: synonymous with love [76,88]; as acts that were seen as violence but were expected, tolerated or justified within relationship norms [45,75,76,87,88,91]; or as violent acts that they rejected and sought to challenge [45,83,89]. Justifications of sexual coercion and force also occurred alongside and did not always preclude desires for love and intimacy [45,76,86,88–90]. Across these responses, children and adolescents described a range of painful and at times contradictory emotions. Authors of studies across diverse settings draw attention to how when adolescent girls and boys negotiate and construct definitions of violence in interpersonal male-female relationships with peers and intimate partners, they do so within the structural gender inequality that underpins such violence and that shapes perceptions of a dominant male sexuality and authority over girls’ bodies [45,75,80,82,84,86,88–90].

#### Age

Children and adolescents’ conceptualisations of violence within included studies were related to age-dependent power dynamics and hierarchies, both inter- and intra-generationally. Boys in South Africa described how physical peer violence was rooted in imbalances of power around age and physical size with older boys, and in some cases girls, holding more power over younger, smaller boys, and girls. This was often reinforced through emotional and physical violence [66,69–71,92]. Children often saw themselves as holding unequal power to adults and at times linked this to violence. This was illustrated in cases of adolescent girls’ vulnerability to violence in relationships with older men [45,77,86] where gender and age inequalities intersected or through perceptions of adult authority over children and adolescents that unpinned several forms of caregiver or institutional adult violence [67,71–73,79,86]. Views on and the types of violence experienced were also dynamic and changed as children grew older. This was also gendered in nature. Older children and adolescents more often questioned physical punishment than younger children as they saw themselves as more independent from adult authority [64,72,79] which some adolescents in Ghana viewed in relation to gender norms [64,72]. Lack of caregiver financial provision was discussed in particular depth by older children and adolescents and was gendered, with some adolescent boys feeling distressed at responsibility for financial provision for themselves and/or others [72,73] and adolescent girls being described as vulnerable to sexual exploitation in the absence of caregiver financial support [45,73–77].

#### Location/Physical space

Location of the act also shaped the ways in which violence was perceived in gendered ways. This was inter-connected with relationships and age. Experiences of violence and maltreatment in school spaces held particular meaning, with inappropriate touching or advances from male teachers to female learners viewed in terms of gendered and institutional power dynamics in the teacher-student relationship [45,75,82–84]. Girls in secondary schools describing unwanted touching from boys around poorly regulated school spaces [75,82,84], with the playground being a space for boys’ physical violence and negotiation of male hierarchies [66,70]. One study exploring boys and bullying in South African township primary schools identified gender-segregated toilets as both a space of vulnerability and safety for girls. Girls used girls’ toilets as a safe space to run to when escaping physical fighting with boys [66]. Private, public, and isolated spaces gave rise to particular perceptions of sexual violence, with a girl being present in a boy’s bedroom seen by some adolescents as both a reason for inability to escape forced sex and also a potential source of blame and shame [45,87]. For some boys this justified forcing a girl into sex [89]. Forced sex due to drugging and alcohol was viewed to take place in unregulated spaces, such as on the street [72] or at parties [86,87], while rape or defilement was perceived to occur when moving in unsafe community spaces [68,71,73,79,81]. Moving in violent neighbourhoods led to boys’ and girls’ ongoing gendered negotiations of relationships, viewing the safety afforded by adult caregivers or proximity to dangerous actors on the street, differentially as they aged into adolescence in South Africa [69,71] and urban Ghana [72]. Burundian adolescents in Uganda described perceptions of risk and neglect in relation to a refugee setting that was different in nature to their home setting [78]. Authors of several studies highlight how space further intersected with interpersonal relationships and gender or age inequality. In one study in a South African township, adolescent girls were reluctant to disclose physical violence from boyfriends as it was seen as a private matter [80], and in one study in Ghana, adolescents experienced extreme violence from caregivers due to the private space afforded to the home [67].

## 4. Discussion

This review has examined what we currently know about how children and adolescents conceptualise acts defined as violence by international definitions in sub-Saharan Africa. It reveals significant limitations in our evidence base. Studies represent only 12 countries in Sub-Saharan Africa, and only 10 of 30 studies contained any data from children below adolescent age, meaning that views of younger children are under-represented. Forms of violence that are not sexual or within sexual relationships are under studied. Few studies explicitly employed child-friendly or participatory approaches and rarely asked participants open-ended questions about their wellbeing and painful emotions. This likely limited the elicitation of children and adolescents’ views on this sensitive topic.

Nonetheless, we identified six themes in how children and adolescents described their experiences of acts internationally recognised as violence. Across these themes we found that when creating meaning, children and adolescents focused less on the act of violence itself and more on their feelings and overall wellbeing as related to the experience. Their feelings about painful or difficult experiences were relational, and linked to relationship to perpetrator, location of the act, and age-related dynamics, all of which shaped the way acts were viewed. These three areas were gendered in nature and interlinked. These findings were consistent with a study in the Philippines, examining child-centred indicators, that found immediate relationships and environments, particularly at the interpersonal and community-level, to be important to children’s understanding of violence [31]. This also resonates with insights from sociological research that emphasises how the social context in which acts of violence occur shapes the meaning of those acts [40].

Our findings on how children and adolescents conceptualise acts internationally recognised as violence suggest that these both overlap with, and are distinct from, the WHO and UNCRC definitions. Adults abusing and neglecting responsibility emerged prominently from children and adolescents’ narratives as particularly emotionally painful. Although neglect is included in the WHO definition of violence, in practice, it is seldom measured in international surveys [93,94], and does not form the basis of any SDG indicators. Neglect is often discussed as difficult to measure comparably across contexts where resource constraints differ greatly [95–98], but this was clearly important to children and adolescents themselves. Less is known about how violating expected roles within relationships is linked to health and social outcomes, especially in resource-poor settings where caregivers’ economic situations may preclude fulfilment of such roles. Similarly, conceptualisations of childhood and appropriate caregiving vary substantially cross-culturally.

Failure or inability of adults to meet their responsibilities to maintain safe institutional environments also emerged as an important theme. Although also not directly measured in international surveys, safe environments and the lack of them are clearly important for children and adolescents, and adults not meeting expectations to maintain these environments was viewed as a transgression. Work to develop child-centred indicators for violence prevention in the Philippines have similarly found that children distinguish between violent acts and unsafe conditions that lead to harm, while also understanding a complex interaction [31]. Further work is needed to understand children and adolescents’ perceptions of safety in different environments. This links with literature on bystander interventions to prevent violence and challenge unacceptable behaviour [99,100] and a small associated qualitative literature on institutionally responsible adults (for example, teachers) and the barriers they may experience to ensuring a safe school environment [101].

Our findings on emotional violence surrounding sex, and in particular, sex outside of socially sanctioned relationships, also stand out. Female participants describe a range of experiences, including shame and humiliation, for many sexual experiences, across a wide continuum from consensual to forced. Girls themselves did not largely view this as violence, which may suggest that social constraint, risk and stigma may characterise many girls’ negotiations of their sexuality. This is further borne out in wider qualitative literature in the SSA region [102–106]. However, shame and humiliation around sex is also not currently measured directly in large-scale surveys.

We also noted children and adolescents describing painful, humiliating experiences that caused them to suffer greatly and that were in line with WHO and UNCRC definitions of violence, but that they themselves did not view as violence. This could be seen among those children who viewed a certain degree of physical punishment as everyday discipline, adolescent women accepting some violence from intimate partners as inevitable or even as acts of love, or girls discussing girls’ responsibility for rape or judging their peers for exploitative sexual experiences. This resonates with theoretical work on everyday violence, examining forms of routine violence that children experience in their everyday lives [107,108], and structural and symbolic violence [109–111], examining how in contexts of entrenched and structural inequality people may blame themselves, or peers, while unequal conditions at the root of violence remain obscured. At the same time, children and adolescents’ heterogenous perspectives and focus on their own feelings showed that they did not straightforwardly accept these experiences. They simultaneously rejected and/or challenged experiences that caused them suffering and sought fulfilling and positive relationships. Our findings point to the importance of this complexity and negotiation of agency, as well as the structural inequality related to age and gender that constrains many children and adolescents’ lives, and the intersecting ways in which it does so for adolescent girls.

This review aimed to understand what we currently know about how children and adolescents conceptualise violence and how this maps on to international definitions. Our findings showed that children and adolescents in these studies perceived violence in ways that were shaped by relationships, location, and age and gendered dynamics. Such perspectives emphasise that violence is multi-dimensional in nature and can take many forms, including everyday or severe physical and emotional acts, structural, and symbolic forms [37,107,108,110,112]. Our findings suggest that children and adolescents did find meaning in focusing on acts of physical, emotional, sexual violence and neglect, as used in international definitions, but that they conceptualised and interpreted these acts through the lens of their relationships, with the central focus on their feelings and wellbeing. This resonates with literature examining the overlaps and opportunities for synthesis between different approaches that tend to forefront either *acts* of violence; the complexity of its structural and social significance [37,38]; and the importance of understanding how violence is felt and experienced by the survivor [107,108]. These findings link with sociological and anthropological research emphasising the social contexts of violence.

### Strengths and limitations

Our findings resonate with work spanning multiple disciplines, rooted in social theory, that draws out how violence is relational and gendered; dynamic and evolving; and dependent on sociocultural contexts for constructing definitions of violence [36–41,113,114]. Our synthesis approach sought the multiplicity of children and adolescents’ perspectives, important for understanding their worldviews [114–116], and in particular for exploring heterogenous experiences of violence in sub-Saharan Africa in ways that go beyond conceptualising ‘lack’ or marginalisation [117–119], or reinforcing gendered discourses of girls’ homogenised vulnerability [120–122].

Our study also has limitations. Despite extensive searching it is possible that some eligible studies were missed. Due to the large number of papers returned through database searches we did not use citation tracking or conduct reference list screening, nor did we include grey literature in the search. However, to do so would likely have elicited further relevant studies. Children and adolescents’ conceptualisations of violence also occur in studies without explicit focus on this, so relevant data in excluded studies may have been missed. While we did not exclude for language, searches were conducted in English and this likely unintentionally excluded studies not written in English. We are also limited by quality of studies included in our review, with insights limited due to lack of child-friendly, participatory approaches, and those using open-ended questions about children and adolescents’ painful experiences. However, these are also important gaps that we highlight through this review. Studies were rooted in a range of disciplinary approaches. Our own study team comprised multi-disciplinary researchers across public health, anthropology, sociology, geography, and education, with a range of experiences living and working in sub-Saharan African settings. As with any qualitative review, it is likely that a different review team would have elicited different insights.

### Implications for practice and research

More primary qualitative research is urgently needed that examines how children and adolescents conceptualise violence in sub-Saharan Africa. Studies are needed from a greater range of sub-Saharan African countries and settings, and which utilise child-friendly and participatory methods. In particular, due to the difficulty of examining violence when children and adolescents do not use this term, studies comparing and contrasting different research methodologies (such as comparing questioning around acts, to questioning around feelings and wellbeing), would be useful. Research on children and adolescents’ conceptualisations of a wider range of violent experiences is needed, as well as research which includes the perspectives of younger children. Given the importance of neglect and lack of protection in resource-poor settings for children and adolescents highlighted in this review, and lack of epidemiological research on these, primary research is also needed to advance conceptualisation and measurement of these topics. Finally, there is a clear need for future research to employ robust child protection mechanisms and to share findings into the efficacy of such approaches, to both meet ethical obligations to child and adolescent participants, and to advance the methodological field of ethical research into violence against children and adolescents.

Epidemiological research into violence should incorporate children and adolescents’ perspectives in the development of quantitative tools, as these do not always overlap with existing measures and important experiences may be being missed [36]. Such research should examine approaches to more effectively capture neglect, safe and unsafe environments, and emotional violence around sex, as these were important for children and adolescents and are not currently widely measured in international surveys.

Further epidemiological research is also needed to examine the health and social implications of our findings. It is unclear whether children and adolescents’ perceptions of relationship context, or cultural context, in which violence occurs influence the associations between these acts of violence and various health outcomes [123]. Children and adolescents perceiving some forms of violence as everyday or acceptable within some relationship contexts, speaks to theoretical approaches into ‘cultural normativeness’ that suggest the consequences of violence may be less severe where it is perceived as normative [124,125]. However, research into the impact of ongoing or repeated trauma suggests that effects of repeated violence may result in physiological wear and tear on our bodies and have cumulative physical effects, even if it is not necessarily experienced as overtly stressful [126–128]. More research is needed in this area. Considering our current lack of knowledge about the social and health impact of acts of violence that children and adolescents define relationally, does not suggest disregarding or reducing international definitions of violence, however, but rather expanding them.

Our findings also suggest that interventions to address violence against children and adolescents may be missing some aspects of their experiences which are important to them, particularly in relation to neglect from important adults, and emotional violence around consensual sex that violates social norms. There is also a need for heightened focus on what constitutes safe and unsafe environments for children, particularly those in which adults do not meet their obligations to provide safety and care. The importance of relationships for children and adolescents’ perceptions of violent acts suggests that violence-prevention interventions should be accompanied by reflection on what constitutes violence within various relationships. Overall, our findings strongly suggest that such intervention approaches should be designed in close relation to children and adolescents’ gender and age, and explicitly address gender and age-related inequalities.

## Conclusions

From a limited evidence base, our findings suggest that children and adolescents’ conceptualisations of violence in sub-Saharan Africa both overlap and contrast with WHO and UNCRC definitions. Children and adolescents focus less on defining acts and more on their emotions and overall wellbeing associated with them. They construct conceptualisations through the lens of their relationships with perpetrators, location of the act, and age, and in ways that are gendered and interlinked. In particular, the concept of neglect and adults failing in their responsibilities was highly important for children and adolescents. These are areas to incorporate in future tools to measure violence of children and adolescents in sub-Saharan African settings. There are several key gaps in current evidence, for example geographical spread across sub-Saharan Africa, a wide range of forms of violence, the views of younger children, and depth of insights through lack of child-friendly methodologies. International public health tools need to incorporate children and adolescents’ views in order to measure the right outcomes for them and to understand their social and health implications, and more high-quality qualitative research addressing the gaps above is needed to support this.

## Supporting information

S1 Appendix PRISMA checklist. (DOCX)

S2 Appendix PsychINFO search strategy. (DOCX)

S3 Appendix Quality assessment tool. (DOCX)
