# Supplementary material for: What do we know about how children and adolescents conceptualise violence? A systematic review and meta-synthesis of qualitative studies from sub-Saharan Africa
Source: PLoS One. 2024 Jul 5;19(7):e0304240. doi: 10.1371/journal.pone.0304240 (PMC11226035; doi:10.1371/journal.pone.0304240)
Supplement: S2 Appendix — (DOCX) [file pone.0304240.s002.docx]

**PsychINFO**

1. exp Child Abuse/ or sexual abuse/
2. infant development/ or early childhood development/ or neonatal development/ or adolescent development/ or adolescent characteristics/ or childhood development/ or developmental age groups/ or developmental stages/ or emerging adulthood/ or exp STUDENTS/ or exp PRIMARY SCHOOL STUDENTS/ or exp HIGH SCHOOL STUDENTS/ or exp KINDERGARTEN STUDENTS/ or exp MIDDLE SCHOOL STUDENTS/ or exp PRESCHOOL STUDENTS/ or exp ELEMENTARY SCHOOL STUDENTS/ or early experience/
3. (child* or adolescen* or boy$1 or boyhood or girl* or teen* or preteen* or pubescen* or prepubescen* or youth* or juvenile* or preteen* or pre teen* or young people* or young person* or early adult* or young adult* or infan* or baby or babies or school* or pupil* or student* or nursery or preschool* or pre school* or underage or minor or partner* or spouse* or peer* or boyfriend* or boy friend* or girlfriend* or girl friend or acquaintance* or non stranger* or nonstranger* or agemate or age mate).tw.
4. 2 or 3
5. rape/ or sex offenses/ or exp incest/ or violence/ or domestic violence/ or intimate partner violence/ or partner abuse/ or exp Battered Females/ or exp Aggressive Behavior/ or exp Aggressiveness/ or punishment/ or physical discipline/ or exp BULLYING/ or relational aggression/ or "spouse abuse".tw. or aggression.tw.
6. pedophilia/ or child neglect/ or abandonment/ or cyberbullying/ or child discipline/ or shame/ or embarrassment/ or (rape$1 or rapist or incest or polyvictim* or poly victim* or pedophil* or paedophil* or "corporal punish*" or bully or bullies or anti bully* or bully victim* or cyberbull* or cybervictim* or neglect* or disciplin* or defile or defilement or overbeat or over beat or beat or beating or "bad touches" or "vulgar words" or "love relationships" or humiliat* or ignoring or ridicule).tw.
7. harassment/ or sexual harassment/ or victimization/ or teasing/ or prostitution/ or human trafficking/ or coercion/ or (((sex* or disturbing or loving) adj2 (violen* or abus* or assault* or attack* or aggressi* or coerc* or maltreat* or victim* or re victim* or offence* or offense* or molest* or harass* or exploit* or teas*)) or "virginity test" or "genital mutilation" or "forced marriage" or "forced prostitution").tw.
8. physical abuse/ or child labor/ or working conditions/ or self-inflicted wounds/ or self-destructive behavior/ or self-injurious behavior/ or self-mutilation/ or ((phys* adj2 (violen* or abus* or assault* or attack* or aggressi* or coerc* or maltreat* or victim* or force or restraint)) or caning or slap* or insulting or "hard labour" or "forced labour" or overwork or denying or spanking or pushing or shaken-baby or "forced abortion" or "self-inflicted violence" or sequestrating or biting or strangling or suffocating or burning or kicking or scratching).tw.
9. emotional abuse/ or verbal abuse/ or social isolation/ or threat/ or ((emotion* or psychologic* or mental or excessive) adj2 (violen* or abus* or maltreat* or mistreatment or isolat* or scaring or threat*)).tw.
10. school violence/ or ((gender or peer or agemate or age mate) adj2 (violen* or abus* or assault* or attack* or aggressi* or coerc* or maltreat* or victim* or harass* or mistreatment)).tw.
11. exp Acquaintance Rape/ or ((intimate partner or domestic partner or partner* or relationship* or spouse* or boyfriend* or boy friend* or girlfriend* or girl friend or date or dating or acquaintance* or non stranger* or nonstranger* or agemate or age mate or age-mate or excessive) adj2 (violen* or abus* or assault* or attack* or aggressi* or coerc* or maltreat* or victim* or mistreatment)).tw.
12. exposure to violence/ or ((witness* or expos*) adj2 (violen* or abus* or assault* or attack* or aggressi* or coerc* or maltreat* or harass* or mistreatment)).tw.
13. 5 or 6 or 7 or 8 or 9 or 10 or 11 or 12
14. 4 and 13
15. 1 or 14
16. Exp Africa/
17. ANGOLA.lo. or BENIN.lo. or BOTSWANA.lo. or Burkina Faso.lo. or Burundi.lo. or Cameroon.lo.or Cape Verde.lo. or Central African Republic.lo. or Chad.lo.or Comoros.lo. or (Democratic Republic of the Congo or DRC or Kinshasa).mp. or (Republic of the Congo or Brazzaville).mp. or Ivory Coast.lo. or Cote d'Ivoire.mp. or Equatorial Guinea.lo. or Eritrea.lo. or Ethiopia.lo. or gabon.lo. or gambia.lo. or ghana.lo. or guinea.lo. or Guinea-Bissau.lo. or kenya.lo. or lesotho.lo. or liberia.lo. or madagascar.lo. or malawi.lo. or mali.lo. or mauritania.lo. or mauritius.lo. or mozambique.lo. or namibia.lo. or niger.lo. or nigeria.lo. or rwanda.lo. or (Sao Tome and Principe).lo. or Senegal.lo. or Seychelles.lo. or Sierra Leone.lo. or South Africa.lo. or South Sudan.lo. or Swaziland.lo. or Tanzania.lo. or togo.lo. or uganda.lo. or zambia.lo. or zimbabwe.lo.
18. 16 or 17
19. psychodiagnostic interview/ or structured clinical interview/ or questioning/ or interviewing/ or interviews/ or exp Qualitative Research/ or ethnography/ or curricular field experience/ or role playing/ or self-report/ or self-evaluation/ or self-monitoring/ or self-perception/ or grounded theory/ or journal writing/ or exp narratives/ or ((("semi-structured" or semistructured or unstructured or informal or "in-depth" or indepth or "face-to-face" or structured or guide) adj3 (interview* or discussion* or questionnaire*)) or (focus group* or qualitative or ethnograph* or fieldwork or "field work" or "key informant" or case study or role play or self report or self-report or diary or context* or list or listing or sort or rank or observ* or narrative or grounded-theory or field-studies or field-notes)).ti,ab.
20. Limit 19 to "qualitative (best balance of sensitivity and specificity)"
21. 15 and 18 and 20 Total results=1084
