# Supplementary material for: What do we know about how children and adolescents conceptualise violence? A systematic review and meta-synthesis of qualitative studies from sub-Saharan Africa
Source: PLoS One. 2024 Jul 5;19(7):e0304240. doi: 10.1371/journal.pone.0304240 (PMC11226035; doi:10.1371/journal.pone.0304240)
Supplement: S3 Appendix — (DOCX) [file pone.0304240.s003.docx]

| CASP quality checklist for qualitative research | Our review |  |  | Ranking:  0 – No  1 – Partially/it is mentioned  2 – Yes/discussed in some detail |
| --- | --- | --- | --- | --- |
|  | **Area** | **Overarching question** | **Notes** |  |
| 1. Was there a clear statement of the aims of the research? | **1. Research aim** | Is there a clear statement of the aims of the research? |  |  |
| 2. Is a qualitative methodology appropriate? |  | | | |
| 3. Was the research design appropriate to address the aims of the research? |  |  |  |  |
|  | **2. Theoretical framework** | Is there evidence of a theoretical framework? | Is there description of theoretical frameworks, or overarching approach to analysis? Do the authors make clear the theoretical perspectives underpinning the study? |  |
|  | **3. Context** | 3. Is the context of the study described? | Is the country (social/political/policy) context described? Is the research setting described (ie. district, town, village, school, community etc.)? Is the context adequately described so reader could assess how to relate to other settings? |  |
| 4. Was the recruitment strategy appropriate to the aims of the research? | **4. Study design: sites and sampling** | 4. Do the authors explain and justify the sampling strategy? | Have the authors described the methods for sampling participants, selecting research settings and why they selected them? Is this rigorous and suited to the aims of the research? Do they describe the group of participants and sites they are working with? |  |
| 5. Was the data collected in a way that addressed the research issue? | **5. Study design: data collection methods** | Do the authors explain and justify the methods used? | Have the authors described the methods for data collection and why they selected them? Are these rigorous and suited to the aims of the research? |  |
| 6. Has the relationship between researcher and participants been adequately considered? | **6. Child-friendly / participatory approach** | Are the methods described child-friendly and participatory? | Do the authors state that finding methods of children's meaningful participation was an aim of methods used? Do the authors describe how the methods are child-friendly? Did they share findings with participants for input? Do they reflect on the pitfalls and power imbalances of working with children and how these were addressed? |  |
|  | **6. Reflexivity** | Is there evidence of researcher reflexivity? | Do the authors reflect on how the study design chosen may have influenced the findings; how their personal characteristics, background and relationship to participants may have influenced their observations and interpretations? |  |
| 7. Have ethical issues been taken into consideration? | **8. Ethics** | Are ethical concerns taken into consideration? | Are the procedures for obtaining informed consent, confidentiality and any ethical challenges described? Were ethical approvals obtained? Do the authors reflect on the ethical considerations of conducting violence research and with children, and explain how these were addressed? |  |
|  | **9. Child protection procedures** | Are there referral procedures in place for child protection? | Do the authors describe how disclosures of abuse by children were handled through the research? Did children receive follow-up care and was this rigorous? |  |
| 8. Was the data analysis sufficiently rigorous? | **10. Data analysis** | Do the authors explain how the data was analysed? | Is there an in-depth description of the process of analysis? Is it clear how themes/findings were derived from the data? To what extent were contradictory data taken into account? Is there evidence of reflexivity in this process? |  |
| 9. Is there a clear statement of findings? | **11. Clear findings backed up by data** | Are findings clearly articulated and *often* supported with raw data? | Do the authors clearly state the findings of the research? Are descriptions of participants' perspectives backed up with raw data often, and is it clear and persuasive how claims were made from this data? Are further arguments backed up with further literature and contextualisation? |  |
| 10. How valuable is the research? |  |  |  |  |
